# Supplementary material for: Are carbon emissions trading and green financial instruments synergistic? -Comprehensive quantitative research based on content analysis
Source: PLoS One. 2024 Mar 7;19(3):e0298601. doi: 10.1371/journal.pone.0298601 (PMC10919601; doi:10.1371/journal.pone.0298601)
Supplement: S1 Data — (ZIP) [file pone.0298601.s001.zip › Data/地级市Z_F工作报告.zip!/╣π╢1⁄2/├»├√╩╨/└┤╫╘╣1⁄2╓┌║┼í╛┬φ┐╦╩2╛▌═°í┐_2008.docx]

各位代表：

我代表市人民政府向大会作政府工作报告，请予审议，并请政协各位委员和其他列席人员提出意见。

2007年政府工作回顾

2007年是本届政府任期的第一年。在中共茂名市委的正确领导下，在市人大及其常委会的监督和市政协的支持下，市政府以邓小平理论和“三个代表”重要思想为指导，以科学发展观统领经济社会发展全局，认真贯彻党的十六大、十七大精神，按照省第十次党代会和市第九次党代会的部署，围绕“建设和谐粤西经济强市、争当东西两翼科学发展排头兵”的目标，团结和依靠全市人民，深入推进改革开放，着力转变发展观念，大力改善发展环境，调整优化产业结构，努力提高发展质量，加强以改善民生为重点的社会建设，全市国民经济保持平稳较快发展，社会各项事业全面进步，人民生活继续改善，较好地完成了市十届人大一次会议确定的各项任务。

——经济综合实力显著增强。全年实现生产总值1066.85亿元，比上年增长（下同）13.1%。其中，第一产业235.83亿元，增长4.8%；第二产业422.08亿元，增长12%；第三产业408.94亿元，增长18.9%，产业结构进一步优化。全市财政预算总收入（不含基金收入）75.7亿元，增长24%；财政一般预算收入31.66亿元，增长23.1%。年末全市金融机构人民币存款余额621.81亿元，比年初增长7.5%。

——工业经济快速发展。工业总体实力和经济效益实现新提高，全年完成工业增加值375.99亿元，增长13.6%。1～11月规模以上工业利润总额53.52亿元，同比增长41.7倍。茂名石化公司、茂名热电厂进入了新一轮快速发展周期，100万吨乙烯工程、6号机组全面建成投产。规模以上工业完成增加值246.51亿元，增长13.1%，其中地方86.36亿元，增长15.5％。产业转移工业园建设步伐加快。全市进入各级工业园区的在建项目132个，总投资21.26 亿元；博贺电厂、北山岭港区30万吨码头一期工程、茂湛铁路等项目的前期工作加快进行。

——农村经济稳步发展。农业基础继续巩固，社会主义新农村建设取得新成绩。全年农林牧渔业总产值354.1亿元，增长4.7%。农业结构进一步优化，粮食、水果、肉类、水产品总产量分别增长7%、4.6%、3.6%和4.2%。支农惠农政策得到落实，农业产业化和农产品质量安全水平不断提高。农业龙头企业带动农户16.36万户，实现销售收入31.36亿元；新增国家级良种场2个、绿色食品认证2个和无公害产品4个。全年减轻农民负担3.6亿元，农资综合直补、粮食直补、能繁母猪补贴等各项补贴资金及时足额发放，农民直接受惠7440万元。

——服务业加快发展。第三产业对GDP增长的贡献率达57.3%，拉动全市经济增长7.5个百分点。全年社会消费品零售总额405.77亿元，增长18.7%。批发零售贸易业、住宿和餐饮业等快速增长，房地产、电信、金融等加快发展。全年商品房销售面积增长31.1%，销售额（含预售）增长60.7%。邮电业务总量增长18.3%。年末金融机构人民币贷款余额232.39亿元，比年初增长6.6%，扭转了2006年的下滑局面。旅游业总收入53.65亿元，增长7.8%。“万村千乡”市场工程深入推进，农村消费环境不断改善。

——企业活力有所增强。全年新登记内资企业301户、外商投资企业39户、私营企业903户。产品质量总体水平稳步提高，新增国家免检产品1个、省名牌产品6个、著名商标3件。企业自主创新能力进一步增强，新增省级高新技术企业6家、省级民营科技企业3家、市级工程技术研发中心3家。

——改革开放迈出新步伐。调整了经济工作机制，成立了市重点项目办和招商引资办并开始有效运作。制定了新一轮市与县（市、区）财政管理体制调整方案，新增财力向县级倾斜。实行了财政下管一级的做法，公共财政体制进一步建立与完善。县级电力企业管理体制改革全面完成，信宜、化州、电白执行新电价后，每年可降低电费负担1.88亿元。茂名大厦等国有商业企业和农信社、烟草管理体制、水管体制等改革有效推进，市城信社开始启动退出市场工作。开放型经济加快发展，全年外贸出口总值3.97亿美元，增长17.3%；实际利用外资5007万美元，增长68.8%；口岸建设管理进一步加强。

——城乡建设展现新气象。永久桥维修加固、油城四至六路和计星路南出口段改造、龙岭小区市政配套设施建设等市政工程如期完成，环市北路改建、新湖和龙湖污水治理工程顺利推进，光华北路灯光夜市顺利搬迁，新建改建一批美化、绿化、亮化工程。土地储备工作实现新突破，市级土地储备大幅增加，闲置土地清理回收工作扎实推进。全年完成交通基础设施建设总投资17.99亿元，新建续建公路1569公里，洛湛铁路茂名段进入铺轨阶段，水东港区3万吨级综合码头工程复工建设。全年建设农村公路1544公里，全市90%村委会已通硬底化公路。高州水库除险加固工程和农田水利基本建设顺利推进。

——社会事业取得新进步。义务教育水平进一步巩固提高，全市110多万名农业户口学生和城镇低保户学生享受了免费义务教育，每年减轻群众负担6.6亿元。农村义务教育学校危房改造工程基本完成。高中阶段教育加快发展，全年新增高中阶段学位1万多个，毛入学率由上年的53.38%提高到59.57%，高考上省大专以上人数连续6年稳居全省第二。职业技术教育快速发展，茂名学院教育和建设水平不断提高。精神文明建设有效推进。人才队伍建设得到加强。文化事业有了新发展，成功举办了“2007茂名石油·荔枝文化博览会”，特色文化发扬光大，基层和群众文化工作得到加强。医疗卫生体系不断完善，城乡医疗救治能力不断提高，综合医院基础设施、社区卫生服务等工作得到加强，乡镇卫生院改革开始启动。群众体育蓬勃开展，竞技体育取得好成绩。完成了省下达的人口控制和计划生育指标。国防动员、民兵预备役和拥军优抚安置工作不断进步。外事、侨务、民族、宗教、殡改、统计、打私、气象、防震、经济研究、市志、档案、驻外联络等工作取得新成绩。老龄、妇女、儿童、助残等事业持续发展。

——民生得到有效改善。进一步落实各项就业扶持政策，全年用于就业支出资金增长73%。新增城镇就业岗位46756个，国企下岗失业人员实现再就业2732人，城镇登记失业率低于3.5%，累计有序输出农村劳动力80多万人次。社会保险覆盖面有所扩大，社保基金征管工作继续加强，社保待遇按时足额发放，企业退休人员人均养老金每月提高了119.2元。城乡最低生活保障制度进一步落实，基本实现应保尽保，市区和农村低保标准有新的提高，对百岁老人的补贴每月增加了200元。农村医疗保障条件有效改善，2007年新型农村合作医疗参合率达81.3%。廉租房建设加快，农村告别泥砖房工程成效显著，我市荣获全国首个“减灾安居工程模范市”荣誉称号。投资5650万元，解决了农村11万多人的饮水不安全问题。安全生产形势稳定，重特大事故得到有效遏制。城乡居民生活水平稳步提高，城市居民人均可支配收入11195元，增长8.9%；农民人均纯收入5051元，增长5%。居民消费价格上涨3.5％。

——环境保护有效推进。积极实施55项节能和资源综合利用项目，加快推广新型墙体材料、散装水泥和农村沼气利用。市区新上项目“环评率”和“三同时”执行率达100%。污水处理厂、电厂脱硫装置建设进展顺利，城镇生活污水集中处理率和城市生活垃圾集中处理率有所提高。林业生态市建设步伐加快，高州水库生态保护工作进一步加强，新设一批自然保护区，全市森林覆盖率达56.4%。海洋生态建设有效推进。全市环境质量保持稳定，生产生活用水和空气质量良好，全年二氧化硫排放量、化学需氧量排放量分别减少1.7%和4.5%。

——民主法制和政府自身建设不断加强。认真执行市人大及其常委会各项决议并向其报告工作，坚持向人民政协通报情况，自觉接受人大监督和政协民主监督。人大代表建议、政协提案办理满意率分别达到99.2%和99.6%。密切与工会、共青团、妇联等人民团体的联系，广泛听取社会各界意见。落实信访工作责任制，信访总量持续减少。深入推进平安茂名建设、公安“三基”工程建设和科技强警示范城市建设，加强公安消防和监狱管理工作，社会秩序保持稳定，各类案件持续下降，没有发生大规模的群体性事件和群死群伤的恶性事故。行政复议、人民调解、司法救助、普法等工作有效推进。加强药品监督，认真开展产品质量和食品安全专项整治。认真实施行政许可法、公务员法和全面推进依法行政实施纲要，深入推进行政审批制度改革，建立行政审批电子监察系统。认真落实党风廉政建设责任制，全面推行政务、厂务、村务公开，政府工作透明度增强，行政监察、审计监督得到加强。政府依法行政、驾驭市场经济、统筹经济社会发展的能力不断提高。

各位代表！过去一年所取得的成绩，是上级党委、政府和中共茂名市委正确领导的结果，是全市人民团结奋斗、开拓进取的结果。在此，我代表市人民政府，向全市人民，向中央、省驻茂单位和驻茂解放军、武警部队官兵，向各民主党派、人民团体以及社会各界人士，表示诚挚的感谢！向所有关心、支持茂名建设的香港及澳门特别行政区同胞、台湾同胞、海外侨胞和国际友人，表示衷心的感谢！

回顾一年来的工作，我们清醒地看到，我市经济社会发展仍然存在不少困难和问题。主要是：经济基础还相当薄弱，整体素质和竞争力与先进地区相比仍有明显差距；固定资产投资总量少，加快发展后劲不足；工业经济依附性强，地方工业规模小，自我发展能力不强；现代农业发展任重道远，农民增收难度大；县域经济依然薄弱，财政收支矛盾突出；基础设施建设、城市规划建设滞后于经济发展需求；用地难、用电难、贷款难问题依然突出；节能减排任务十分艰巨；社会事业发展水平有待进一步提高；物价上涨偏快，民生领域还存在一些亟待解决的问题；政府机关大局意识、服务意识有待提高，作风亟需转变；全社会的创业意识、危机意识、忧患意识有待进一步增强。对这些问题，必须认真采取措施加以解决。

2008年工作安排

今年是全面落实党的十七大精神的第一年，是以新一轮思想大解放推动新一轮大发展的重要一年。当前，我市经济社会发展态势良好，具备了加快发展的物质基础、产业基础和外部条件。省委、省政府推进现代产业体系建设、加快推进区域协调发展等一系列举措，为我市创造了难得的发展机遇。我们必须按中央、省的部署和要求，深入贯彻落实科学发展观，牢牢把握发展机遇，准确定位发展方向，解放思想，锐意创新，努力推动经济社会又好又快发展。

今年经济社会发展的主要预期目标：生产总值增长11%，单位生产总值能耗降低3.2%，全社会固定资产投资增长18%，社会消费品零售总额增长14%，外贸出口增长10%，实际利用外资增长15%，地方财政收入与经济同步增长，居民消费价格涨幅控制在4%左右，城镇登记失业率控制在3.3%以内，城镇居民人均可支配收入增长8%，农村居民人均纯收入增长6%，人口自然增长率控制在7.5‰以内。

省委、省政府提出，今后五年全省必须抓好加快转变经济发展方式，加快完善社会主义市场经济体制这“两个关键”；“坚持六个突出”：突出抓好自主创新、突出抓好现代产业体系建设、突出抓好改革开放、突出抓好节能减排和生态建设、突出抓好城乡区域协调发展、突出抓好以改善民生为重点的社会建设。这也是茂名今后几年发展的总体要求。做好今年政府工作，完成我市今年发展目标，逐步达到总体要求，解放思想是前提，创新思路是关键，深化改革是动力，转变作风是保障。我们要按照市委九届四次全会的要求，在“解放思想、创新思路、深化改革、转变作风”这四个方面下功夫。同时，还必须把握“重基础、激活力、抓落实、优发展、促和谐”的工作方针。重基础就是要牢固树立基层是重点、基层是基础的观念，把更多的资源投向基层，推动县镇发展，夯实我市发展之基；要高度重视规划的基础性作用；要加大对基础设施建设的投入，优化我市的发展环境。激活力就是要进一步营造干事创业的良好氛围，形成公平竞争的良好局面，外引内联结合，激发全民创业活力。抓落实就是要踏踏实实，重实际、鼓实劲、求实效，凡事周密计划部署，精心组织实施，严格督促检查。优发展就是要注重发展的质量、发展的效益和发展的可持续性，努力平衡当前利益和长远利益，增强发展后劲。促和谐就是要高度重视民生，倾听民意，纾解民困，改善民生，共建共享发展成果；采取措施促进各县（市、区）均衡发展；适度、适量利用自然资源，进一步加强环境保护，促进人与自然和谐共处。今年要重点推进十一方面的工作：

一、突出发展沿海石化产业，推进海洋经济建设

抓好载体建设，打造良好的发展平台。高起点、高标准规划和建设北山岭港区。加快石化工业区建设，突出解决征地等难题，提高园区对项目的承载能力。结合改善人居环境，利用现有公用工程基础，整合市区河西片适用产业发展用地，推动相关工业项目和物流业的合理布局。加强各级各类工业园区的基础设施建设，提高管理服务水平，进一步增强园区对各生产要素的吸引力。

积极构筑以大企业为龙头，大中小企业错位协调发展的格局。大力支持加快2000万吨炼油等重大项目的建设，为茂名石化公司巩固全国最大炼化一体化企业地位、增强“龙头”辐射和带动力创造有利条件。鼓励支持龙头骨干企业推进自主创新，加快产品升级换代，衍生更多的石化后加工企业。引导关联产业、企业、项目与龙头企业链接。鼓励支持民间资本参与石油化工、乙烯后续加工及下游产品配套开发利用，延伸产业链，形成相互配套的“簇群经济”。

推进海洋经济建设。充分利用得天独厚的海洋资源，以北山岭港区建设和争取设立国家级博贺渔港为契机，扎实推进海洋经济建设。加大港口基础设施建设力度，大力发展海洋交通运输、海洋渔业、滨海旅游、海洋船舶、海洋化工和海洋生物医药等。鼓励民间资本参与海洋综合开发利用，积极扶持高新技术企业开展项目研究与建设，有序开发海洋资源，努力使海洋经济成为我市重要的经济增长点。

二、加快县域经济发展步伐

大力发展县域工业。认真落实鼓励县域经济发展的政策措施，调动各县（市、区）的积极性。大力发展石化产品后加工、农副产品深加工、海洋资源加工工业，壮大特色工业。抓好矿产资源的统筹规划及开发利用，努力提高矿产资源开发的综合效益。继续做好产业转移承接工作。扶持带动能力强、拥有自主知识产权的重点行业、重点企业和重点产品。推进信息化与工业化融合，用高新技术、先进实用技术和信息技术改造提升特色产业，增强产业竞争力。以工业园区和中心镇、专业镇为主要载体，引导工业项目集聚，实现集约化、规模化发展。

推进现代农业建设。加强农业基础设施建设，加快高州水库及其他水库除险加固和灌溉工程建设，为现代农业发展奠定良好的基础。加大农业科技投入，着力建设带动效应好、竞争力强的现代农业园区，倡导农业标准化、品牌化生产。发展优质粮食生产，改善水果、北运菜等品种结构，培育新的优势特色产品。大力发展市场需求量大、经济效益高的现代养殖业。加快现代林业发展，大力发展水源林、生态林。加强动植物疫病防控和农产品质量监管。加快推进农业产业化经营，发展壮大农业龙头企业，引进培育一批农产品加工企业，推进农产品精深加工，提高产品附加值，增加农民收入。今年市级财政用于农林水事务的各项支出将比去年预算增长67.9%。

改善农村公共服务。继续加强“三农”工作，落实各项强农惠农政策，加大对农业农村发展的投入，着力解决农民生产生活中最迫切的实际问题，扩大公共财政覆盖农村的范围，推动新农村建设深入发展。加强基本农田的保护和标准化改造。继续抓好农村告别泥砖房工程。加强农村规划建设管理，推进“五改”工程建设，实施农村“能源工程”，推广沼气和太阳能利用，推动生态文明村建设，不断改善农村面貌。

三、做大做强民营经济

抓住新一轮思想大解放的契机，广泛开展全民创业宣传活动，增强全民创业意识。进一步完善和落实促进民营经济发展的政策措施，努力形成政府鼓励创业、社会支持创业、民众自主创业的发展环境。加强和改进服务，加快信用体系建设，推进银企合作，帮助中小民营企业解决实际困难。引导企业建立和完善现代企业制度，建设技术创新中心，加快发展自主品牌，增强企业竞争力。积极实施“回归工程”，鼓励外出人员回乡创业，增强民营经济发展后劲。

四、推进重点项目建设

加快项目建设进度。今年拟安排新开工和续建的重点项目

46项，总投资305亿元。进一步提高对重点项目的服务水平，优化项目建设环境。执行重点项目情况报告和督办等制度，及时协调解决项目建设的实际问题和困难。确保洛湛铁路、茂湛铁路、茂名热电厂7号机组、油页岩发电项目等重点项目顺利推进，争取早日建成投产。

抓好项目前期工作。加强项目信息收集整理，科学谋划可行项目，及时调整、充实项目库。抓好重点项目的策划和包装工作，提高项目吸引力。跟踪落实签约项目和有意向项目，实行“一站式”办公和“一条龙”服务，为项目报审创造条件。

落实项目建设保障。积极探索融资模式，努力争取上级资金用于重大项目建设，鼓励民资参与重点项目建设。抓好土地征管、储备和经营，解决项目建设用地问题，引导企业节约集约用地。认真执行项目法人责任制等各项投资管理制度，提高项目建设水平。

五、大力发展现代服务业

优化服务业发展环境。制定促进服务业发展的政策措施，扩大服务业对外开放领域。坚持统筹协调、分类指导，构建充满活力、特色明显、优势互补的服务业发展格局。鼓励和引导各类社会资本进入服务业领域，培育壮大服务业市场主体。规范服务市场秩序，加强行业监管，维护消费者合法权益。

巩固发展传统服务业。大力推进旅游景区景点和高档酒店建设，整合旅游资源，培育特色旅游产品，提升旅游业发展水平。严格执行国家对房地产业的宏观调控政策，规范房地产中介服务，适度引进实力强、信誉好的房地产企业开发大型楼盘，推进住宅产业化和集约化，促进房地产业健康有序发展。

加快发展生产性服务业。以中心城市、县城和中心镇为载体，以争取建设国家级农产品交易市场为突破口，高起点规划建设生产性服务业集聚区，加强物流基础设施规划和建设，完善物流服务体系，形成规模大、层次多、辐射广的工农业服务基地。重点推进专业市场、信息、仓储、金融、科技和商务服务等现代生产服务业，积极发展保险、会计、律师、租赁、文化体育、社区服务等新兴服务业，拓展发展空间，推动服务业加快发展。

六、进一步提高对外开放水平

加强招商引资。健全招商引资工作机制，突出重点，积极向外推介塑料加工、精细化工、农产品深加工、港口基础设施建设、物流等重点招商项目。在招商引资过程中，从不求所有、但求所在、追求所得出发，做到有选择、有取舍，大力引进符合环保要求、有利于我市加快发展的高效益项目，提高招商引资质量。

不断提高对外贸易水平。推动皮手套、竹编、纺织、铸铁等传统外向型产业优化升级，扶持壮大运动器材、精细化工、农产品加工等新兴产业群，大力培育和发展新的出口增长点。支持民营企业开拓国际市场，鼓励企业创建出口品牌，提升国际竞争力。积极推动对外经济合作，支持企业开展境外投资。加强口岸建设管理，提高通关效率。

七、推动生态文明建设

抓好节能减排。严格落实节能减排工作责任制，强化执法监察。鼓励发展低能耗、高附加值的产业，严格限制新上高消耗、高污染项目。开发推广能源资源节约和综合利用新技术、新工艺、新设备、新产品，积极引导重点领域、重点行业、重点企业做好节能减排工作。大力发展新能源和可再生能源，提高能源资源利用效率。推行清洁生产，加快推进发展循环经济。推广政府绿色采购，强化全社会节约环保意识。

加强环境保护和生态建设。切实做好主要污染物排放总量控制工作。严把审核、监管两个闸门，提高环保准入门槛。认真做好污染源普查工作，强化环境污染综合整治，严肃查处污染环境的违法行为。抓好环保基础设施建设，加快茂名污水排海总管工程、高州水库库区生态保护工程、市区河西和各县（市）污水处理厂、垃圾发电厂和填埋场等的规划和建设。加大对固体废物回收和利用处理力度。推进大气污染防治，减少废气排放。加强饮用水源、海洋生态环境和生物多样性保护。抓好大雾岭、河尾山等自然保护区建设，积极创建林业生态市。

八、加强城乡建设与管理

加强城乡规划工作。加快编制新版《茂名市城市总体规划》，抓紧完成《茂名市城镇规划》和有关专项规划的编制工作。积极推进国土规划和土地利用规划修编工作。用超前的眼光科学规划环水东湾新城和茂名大道以东地块，拉大城市框架，加快城市向东向南临海发展，拓宽城市发展空间。贯彻实施《城乡规划法》，按“省管到县，市管到镇，县管到村”的规划管理要求，抓好各县（市）城区和镇、村规划与整治。

统筹城乡建设。积极推进茂名大道的改造与建设，启动市区到海边几条主干道的立项与建设。加快城市道路的建设与改造，完善市政公用配套设施，搞好市政园林绿化，改善城市人居环境。做好迎接“国家园林城市”复查的准备工作。加快县城、中心镇和重点小城镇建设步伐，提高城镇综合承载能力。抓好村庄整治，推进社会主义新农村建设。

创新城市管理方式。加大闲置土地回收力度，推进节约集约用地，增强政府对城市土地开发和利用的调控能力。转变城市经营理念，创新城市管理模式，提高城市的品位和吸引力。加强城市社区建设管理。实行规划设计招标，引进高水平规划设计队伍和人才，逐步提升我市城乡规划设计水平。积极稳妥推进城市管理体制改革，进一步理顺关系、转变职能，完善城市管理体制。

九、完善财政管理体制

坚持依法治税，强化税收征管，加强非税收入征收管理，严格实行“收支两条线”。积极应对税制改革，推进财政管理体制改革，认真实施新的财政管理体制方案，完善县、镇两级激励型财政体制。继续扩大部门预算、国库集中收付范围，推进市级财务核算集中监管改革试点和公务卡试点工作。进一步完善公共财政体系，调整优化财政支出结构，完善支出绩效评价。全面实施行政经费下管一级的制度，加大对各县（市、区）工资专项资金的监管，确保工资及时足额发放。增加对“三农”、教育、医疗、社保、就业等方面的支出。加大转移支付力度，增加对基层的投入，今年市级财政补助县（市、区）的支出将达到2.75亿元，占总支出的13.7%；为解决镇级政府工作运转困难，增强基层政府提供公共服务的能力，今年给全市每个镇补助10万元。

十、大力发展民生事业，促进社会和谐

促进社会充分就业。建立促进就业目标责任制和政府财政投入的保障机制，推动以创业带动就业，多渠道开发就业岗位。建立健全就业援助制度，重点帮助城镇零就业家庭、低保家庭、农村贫困家庭、被征地农民就业和大中专毕业生就业。全年新增城镇就业岗位4.75万个。抓好实施百万农村青年、退役士兵等职业技能培训工程，加快农村劳动力转移。贯彻落实《劳动合同法》，规范各类用人单位劳动用工制度，维护职工合法权益。强化劳动保障监察执法和劳动争议仲裁工作，促进劳动关系和谐稳定。

完善社会保障体系。加大社会保险扩面征缴力度，推进社会保险市级统筹，逐步做实个人账户。加强社会保障基金的筹措、管理和运营。完善最低工资保障制度，建立企业职工工资正常增长机制和支付保障机制，逐步提高企业退休职工待遇。规范完善医疗、失业、生育、工伤保险制度。全面推进城镇居民基本医疗保险试点工作，提高新型农村合作医疗保障水平。健全社会救助体系。落实“低保”、“五保”政策，集中供养的五保户供养标准提高到每户每月200元，分散供养的五保户供养标准提高到每户每月100元。筹建市级孤儿院。鼓励和支持慈善事业发展。探索和建立被征地农民生产生活保障和社会养老制度，逐步健全农村养老保险制度。为加快社会保障体系建设，今年市财政将安排2.24亿元，比去年预算增长35.8%。完善住房公积金制度，扩大公积金归集覆盖面，增强中低收入职工住房有效需求。加快廉租住房建设，着力解决城市低收入家庭住房困难。

大力发展教育文化卫生等事业。巩固农村免费义务教育，实施城镇免费义务教育。加强中小学校校舍建设，逐步解决市区学校大班额问题，努力增加高中学位。大力发展职业技术教育，促进和规范民办教育事业发展。扶持和促进高等教育加快发展。今年市级财政将安排教育经费3.22亿元，比去年预算增长38.3%。推进文化体制创新，增强文化事业发展活力和文化产业发展动力。加强基层文化建设和文化市场管理，发展壮大文化产业。具有公益性质的博物馆、纪念馆和爱国主义教育示范基地，今年内实现全部向社会免费开放。坚持公共医疗卫生的公益性质，完善覆盖城乡居民医疗保障制度，推进城乡卫生服务体系建设，加大力度推进薄弱乡镇卫生院改造，发展城市社区卫生服务，提高城乡医疗服务水平。完善疾病防控体系。加强卫生监督执法。推进中医药强市建设。今年市级财政将安排1.01亿元用于医疗卫生事业，比去年预算增长39.1%。大力发展群众性体育活动和竞技体育，推动体育产业发展壮大。

抓好安全生产、产品质量和食品药品安全等工作。严格落实各级政府安全生产监管主体责任和企业安全生产主体责任，加强“双基”建设，强化监管，深入开展各项安全生产专项整治，坚决防范和遏制重特大事故发生。坚决打击制售假冒伪劣产品和传销行为。加强食品药品监督体系建设，严格食品药品的市场准入，强化监管，确保人民生命安全。加强市场和物价监管，抓好粮、油、肉、菜等生活必需品的供给，努力防止物价过快上涨。

加强人口计生和社会管理工作。加强人口计生新机制建设，突出计生工作重点，落实各级政府计生工作责任制，稳定低生育水平，综合治理出生人口性别比偏高问题，提高人口素质。继续抓好殡葬改革，推进绿色殡葬。做好信访和社会稳定工作。落实各项民生政策，努力为群众办实事、办好事、解难事，让广大人民群众共享改革发展成果。严厉打击各种违法犯罪活动，推进“平安茂名”建设，促进社会和谐稳定。继续抓好国防动员、民兵预备役和拥军优属工作。加强对互联网虚拟社会的管理。抓好村级换届选举。继续统筹做好审计、统计、外事侨务、民族宗教、对台、地方志、档案、气象、地震、贸促和红十字会等工作,推动社会全面进步。

十一、切实加强政府自身建设

围绕建设行为规范、公正透明、勤政高效、清正廉洁政府的目标，进一步转变政府职能，创新运作和管理机制，着力加强政风建设。

转变政府职能。加快推进政企分开、政事分开、政府与市场中介组织分开，完善经济调节和市场监管，努力创造良好政务环境。强化社会管理和公共服务，优化公共资源配置，改进服务方式，大力建设服务型政府。加强应急管理体系建设，提高预防、应对自然灾害和处置突发公共事件的能力。优化政府机构设置，推进事业单位分类改革。

创新政府运行机制，努力形成决策科学、执行顺畅、监督有力的工作机制。完善行政协调和决策机制，合理划分决策主体权限，提高政府执行力。完善重大事项调查研究、专家咨询、社会公示与听证、集体决策等制度，建立健全决策责任制度，使政府决策更加科学有效。坚持依法行政，依法管理经济社会事务，建设法治政府。加强行政监察工作，落实执法责任制和过错追究制度，规范行政执法行为。自觉接受人大监督，认真落实人大及其常委会的决议、决定，积极支持人民政协履行职能，提高议案、建议和提案办理质量。广泛听取各民主党派、工商联、无党派人士的意见和建议，支持工会、共青团、妇联等人民团体更好地发挥作用。健全审计监督和层级监督，加强行政复议工作。

推进政府管理创新。改善投资服务管理，真正实行“一站式”服务，提高服务质量与效率，更好地为企业和公众服务。深化行政审批制度改革，继续精简和规范行政审批。加快电子政务、行政审批电子监察系统建设。深入推进政务公开，提高政府工作透明度，建设“阳光”政府。实行政府绩效管理制度和行政问责制度，提高行政效能。强化行政成本效益观念，降低行政成本，建设节约型政府。

加强政风建设。按照“为民、务实、清廉”的要求，深入推进机关作风建设，营造积极向上的良好氛围。认真开展政府系统解放思想学习讨论活动，破解影响发展的难题，探索加快发展的新路。加强公务员管理，从严治政，确保政令畅通、工作落实。增强机关工作人员大局意识、责任意识和服务意识，切实服务基层、企业和群众。继续精简会议和文件。抓好廉政建设和反腐败斗争，坚决纠正损害群众利益的不正之风。

　 各位代表，做好今年的工作意义重大。让我们共同努力，以科学的态度和务实的精神，改革创新，奋发有为，更好地推动茂名科学发展、和谐发展！
